# The role of the cytosolic HSP70 chaperone system in diseases caused by misfolding and aberrant trafficking of ion channels

**DOI:** 10.1242/dmm.014001

**Published:** 2014-03

**Authors:** Jason C. Young

**Affiliations:** McGill University, Department of Biochemistry, Groupe de Recherche Axé sur la Structure des Protéines, 3649 Promenade Sir William Osler, Montreal, QC H3G 0B1, Canada

**Keywords:** Chaperone, Cystic fibrosis, Long QT syndrome, Degradation, Intracellular trafficking, Protein folding

## Abstract

Protein-folding diseases are an ongoing medical challenge. Many diseases within this group are genetically determined, and have no known cure. Among the examples in which the underlying cellular and molecular mechanisms are well understood are diseases driven by misfolding of transmembrane proteins that normally function as cell-surface ion channels. Wild-type forms are synthesized and integrated into the endoplasmic reticulum (ER) membrane system and, upon correct folding, are trafficked by the secretory pathway to the cell surface. Misfolded mutant forms traffic poorly, if at all, and are instead degraded by the ER-associated proteasomal degradation (ERAD) system. Molecular chaperones can assist the folding of the cytosolic domains of these transmembrane proteins; however, these chaperones are also involved in selecting misfolded forms for ERAD. Given this dual role of chaperones, diseases caused by the misfolding and aberrant trafficking of ion channels (referred to here as ion-channel-misfolding diseases) can be regarded as a consequence of insufficiency of the pro-folding chaperone activity and/or overefficiency of the chaperone ERAD role. An attractive idea is that manipulation of the chaperones might allow increased folding and trafficking of the mutant proteins, and thereby partial restoration of function. This Review outlines the roles of the cytosolic HSP70 chaperone system in the best-studied paradigms of ion-channel-misfolding disease – the CFTR chloride channel in cystic fibrosis and the hERG potassium channel in cardiac long QT syndrome type 2. In addition, other ion channels implicated in ion-channel-misfolding diseases are discussed.

## Introduction

Diseases caused by defects in the folding or trafficking of cell-surface ion channels demonstrate how genetic lesions lead to problems at the biochemical and cellular levels, with clinical consequences. At the genetic level, such diseases arise from single missense or, less commonly, nonsense or frameshift mutations in individual genes, causing loss of function. However, the genetic lesion often affects associated domains rather than the active sites of the encoded proteins, causing protein misfolding at the biochemical level, as opposed to loss of ion-channel function per se. This misfolding causes, at the cellular level, an inability to traffic from the endoplasmic reticulum (ER) to the plasma membrane (PM), and leads to subsequent clearance of the misfolded proteins by the ER-associated degradation (ERAD) system. The cytosolic domains of ion channels can be assisted in their folding by an extensive system of molecular chaperones in the cytosol. At the same time, some of these chaperones also help select misfolded domains to be degraded (Goldberg, 2003; Hartl et al., 2011).

On the basis of this dual role, molecular chaperones could represent a therapeutic target unique to diseases caused by the misfolding and aberrant trafficking of ion channels (referred to here as ion-channel-misfolding diseases). Enhancing the ability of chaperones to assist folding might at least partially rescue trafficking of the mutant proteins, and consequently increase function at the PM. Furthermore, reducing chaperone involvement in ERAD might increase the population of mutant proteins available to be rescued. Finally, enhanced chaperone-mediated ERAD might relieve the dominant-negative toxic effect of mutant subunits that are part of oligomeric channels. In principle, manipulation of the chaperone system alone could help alleviate the multiple physiological defects and clinical symptoms of ion-channel-misfolding diseases.

Perhaps the best-studied example within this class of diseases is the channelopathy cystic fibrosis (CF), which is caused by mutation of the cystic fibrosis transmembrane conductance regulator, CFTR (*ABCC7*). CFTR is a chloride channel that plays an important role in the maintenance of ion balance and thus hydration of epithelial surfaces, most prominently in the lung airways and pancreas. CF alleles are recessive, and affected individuals carry disease mutations in both copies of the *CFTR* gene. The most common CF allele encodes a single ΔF508 mutation in CFTR and, although many other CF mutations are now known, the great majority of individuals with CF are homozygous or heterozygous for ΔF508 CFTR (Davis, 2006; Riordan, 2008). The ΔF508 mutation disrupts the folding of the channel in its cytosolic regions (Riordan, 2008; Lukacs and Verkman, 2012). Loss of CFTR function as a result of improper folding leads to abnormal, highly viscous mucus secretions in these tissues. In the lung, the thickened mucus obstructs airways and greatly increases susceptibility to infection, in particular by the opportunistic bacterial pathogen *Pseudomonas aeruginosa*. Blockage of the pancreatic ducts impairs secretion of digestive enzymes, resulting in nutritional deficits (Davis, 2006; Riordan, 2008). The overall European and North American prevalence of CF is estimated at 1 in 12,000, with birth incidence as high as 1 in 2000 (Farrell, 2008). Current treatments are directed at the various symptoms: clearing airways, countering infections, and managing malnutrition and inflammation (Davis, 2006). Certainly, therapies directed at addressing the misfolding defect in the most common CFTR mutant, and other mutant forms, by targeting the associated chaperone system would simplify the clinical approach. Yet, as discussed below, how the HSP70 (70 kDa heat shock protein) system relates to CFTR structure and trafficking is still being explored.

Another model of chaperone involvement in ion-channel-misfolding diseases is provided by congenital long QT syndrome type 2 (LQT2), caused by mutation of a voltage-gated delayed rectifier potassium channel, hERG (human ether-a-go-go-related gene), also known as Kv11.1. The hERG channel, which is encoded by the *KCNH2* gene, is most highly expressed in cardiac muscle, where it has a key role in the final phase of repolarization following depolarization-induced muscle contraction. Defects in hERG result in delayed repolarization (observed as long QT interval in electrocardiogram), with elevated risk of arrhythmia, ventricular fibrillation and cardiac arrest (Sanguinetti and Tristani-Firouzi, 2006). The prevalence of LQT2 is 1 in 5000–10,000. Unlike CF, which is recessive, congenital LQT2 is dominantly inherited; moreover, there is no single predominant LQT2 mutation in hERG. Of the many disease alleles, some affect channel activity without affecting trafficking, whereas others impair trafficking due to misfolding of hERG. Current preventive treatments, such as reducing β-adrenergic signaling, activation of which can trigger arrhythmia, are only moderately effective (Sanguinetti and Tristani-Firouzi, 2006; Lu and Kass, 2010). It is feasible that therapies that rescue the defective trafficking and function of mutant hERG, the underlying cause of the syndrome, will have a greater preventive effect.

The interconnected HSP70 and HSP90 cytosolic chaperone systems (Kampinga and Craig, 2010; Hartl et al., 2011) have been broadly implicated in folding-dependent trafficking of CFTR and hERG (Loo et al., 1998; Ficker et al., 2003). The role of HSP90 was initially established by the use of specific inhibitors, but this approach was not available for HSP70. However, recent progress has been made through studies of regulatory co-chaperones of HSP70, some only newly identified, and by gene silencing. There might be more complexities associated with HSP70 function than previously thought. Here, the roles of HSP70 in the folding and degradation of CFTR and hERG will be discussed, comparing the wild-type (WT) and mutant forms of the channels, with a focus on recent work. Additional examples of HSP70 involvement in trafficking channels via the secretory pathway will be surveyed, but only where functional evidence is available – HSP70 is one of the most commonly identified interactors in proteomics screens, yet it is often not followed up experimentally. Further validation of HSP90 involvement, moving beyond the earlier inhibitor studies, will be discussed where relevant to understanding the HSP70 mechanisms. To begin with, an overview of the HSP70 system and its associated co-chaperones is provided.

## The HSP70 mechanism

### ATPase cycle

**Fig. 1.**
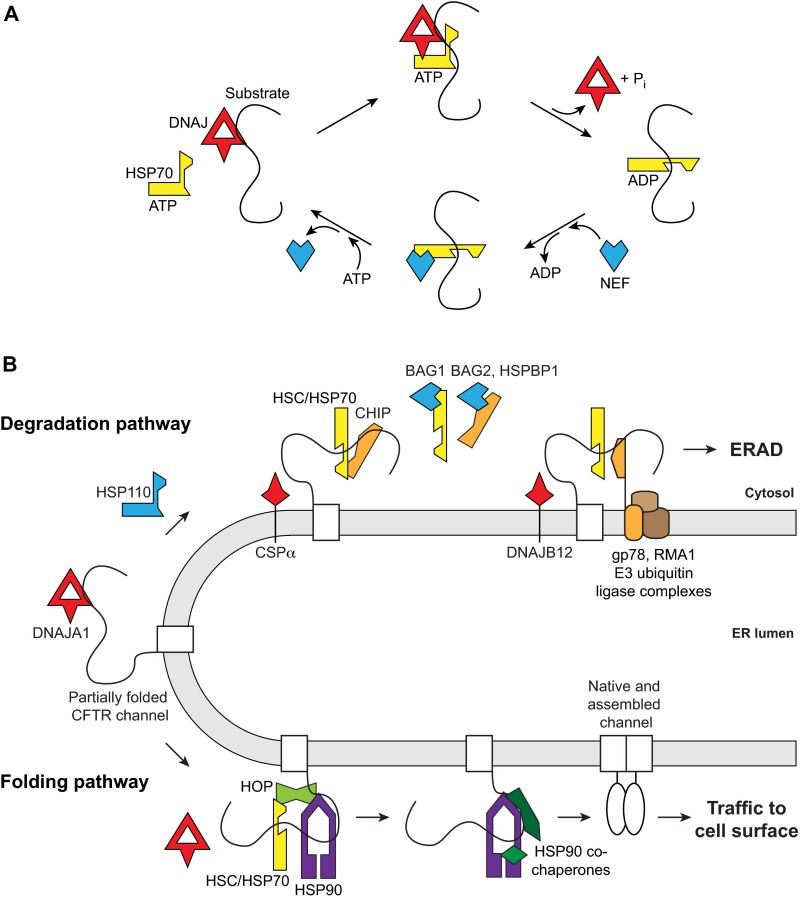
**Mechanisms of HSC/HSP70 functional interaction with substrate polypeptide**. (A) The HSP70 ATPase cycle. Clockwise, starting from left: in the ATP-bound state, HSP70 does not bind substrate polypeptide. A substrate-binding DNAJ co-chaperone contacts HSP70 to stimulate ATP hydrolysis. In the ADP-bound state, HSP70 binds substrate tightly, and the DNAJ dissociates. An NEF co-chaperone promotes the exchange of ADP for ATP and dissociates, returning HSP70 to the ATP-bound state. (B) Chaperone-assisted folding and ER-associated degradation (ERAD) of CFTR. CFTR follows chaperone-mediated pathways for degradation (top) as well as folding (bottom). In the folding pathway, starting from the left, DNAJA1 (red) activates the binding of HSC/HSP70 (yellow) to CFTR to initiate folding; co-chaperone HOP (light green) transfers CFTR from HSC/HSP70 to HSP90 (purple) and its co-chaperones (dark green) to complete folding and allow trafficking to the cell surface. In the degradation pathway, the HSC/HSP70 co-chaperone CHIP (orange) is an E3 ubiquitin ligase that promotes degradation of misfolded CFTR. CHIP functions in parallel to membrane-anchored E3 ubiquitin ligases gp78 and RMA1 and associated components (brown), which do not depend on HSC/HSP70. The DNAJ co-chaperones (red) CSPα and DNAJB12 promote CFTR degradation by CHIP, and by gp78 and RMA1, respectively. The NEFs (blue) BAG2, BAG1 and HSPBP1 interfere with CHIP activity, BAG1 by causing HSC/HSP70 to release substrate, BAG2 and HSPBP1 by binding directly to CHIP. The NEF HSP110 also promotes CFTR degradation. HSP110 is homologous to HSC/HSP70 in the ATP-bound state.

In the human cytosol, the major HSP70 chaperones are the constitutively expressed heat shock cognate protein HSC70 (*HSPA8*) and stress-inducible HSP70 (*HSPA1A* and *HSPA1B*), which are very closely related and share an ATP-dependent mechanism conserved throughout evolution. Other cytosolic HSP70s – HSP70-t (*HSPA1L*), HSP70-2 (*HSPA2*) and HSP70-B′ (*HSPA6*) – are tissue-specific, or only expressed upon stress (Daugaard et al., 2007). In the ATP-bound state, the monomeric HSP70 cannot bind substrate polypeptide stably, whereas, in the ADP-bound state, the substrate is tightly bound (Fig. 1A). Two classes of co-chaperones regulate the HSP70 ATPase cycle: DNAJ co-chaperones (homologs of *Escherichia coli* DnaJ) stimulate ATP hydrolysis and substrate binding by HSP70 through a conserved J domain; and the nucleotide exchange factors (NEFs) promote the release of ADP from HSP70, re-binding of ATP and dissociation from substrate (Fig. 1A). Multiple DNAJ-stimulated cycles of HSP70 binding promote polypeptide folding, sometimes in cooperation with other chaperone systems (Palleros et al., 1993; Szabo et al., 1994; Mayer and Bukau, 2005; Kampinga and Craig, 2010; Young, 2010; Hartl et al., 2011).

### Co-chaperones of HSC70 and HSP70

Up to 35 DNAJ and ten NEF human co-chaperones are available to interact with HSC70 and HSP70; however, many are in low abundance or are expressed in only a subset of tissue types. In cases when HSC70 and HSP70 were experimentally compared, most co-chaperones interacted with either chaperone, which is consistent with a shared mechanism (Kampinga and Craig, 2010). The DNAJs are essential for HSC70 and HSP70 to promote folding (Terada et al., 1997; Höhfeld et al., 1995; Tzankov et al., 2008). Some DNAJs bind to substrates themselves, contributing to the substrate folding mechanism of the common HSP70 chaperones. The best-characterized substrate-binding DNAJs are HSP40 (also known as HDJ1 and DNAJB1), HDJ2 (DNAJA1) and DNAJA2 (encoded by *DNAJB1*, *DNAJA1* and *DNAJA2*, respectively) (Mayer and Bukau, 2005; Young, 2010). Other DNAJs do not bind substrates directly, but can be directed to substrates through protein complexes or sites on membranes; many such DNAJs have biological functions that are distinct or even unrelated to folding (Ungewickell et al., 1995; Magga et al., 2000; Young et al., 2003; Dai et al., 2005b). Therefore, the function of each DNAJ must be determined on a case-by-case basis.

All DNAJs have a J domain (DnaJ homology domain) that contacts an HSP70, activating it to hydrolyze ATP and bind substrate. DNAJs are further classified by degree of similarity to *E. coli* DnaJ. The DNAJAs are homologous throughout their sequences, and generally promote folding; DNAJBs are conserved only in their J domains and adjacent regions, and, although some support folding, many do not, instead having other activities related to misfolded proteins; DNAJCs are divergent beyond their J domains and mostly have specialized functions (Caplan et al., 1993; Ohtsuka and Hata, 2000; Qiu et al., 2006; Sahi and Craig, 2007; Hageman et al., 2011; Kampinga and Craig, 2010). Relevant to this Review, DNAJB1 was an early discovery and considered to be a key component in protein folding; however, data, including those on CFTR below, suggest that it might be less active than DNAJA1, which was discovered later. The DNAJ gene names are used here unless the common protein names are more readily recognized.

The NEFs can enhance or inhibit folding through mechanisms that remain poorly understood hitherto (Höhfeld et al., 1995; Höhfeld and Jentsch, 1997; Dragovic et al., 2006; Tzankov et al., 2008). Structurally, the human NEFs belong to three unrelated families: the BAG family [homologous to BAG1 (Bcl2-associated athanogene 1)], the HSP110 family and HSPBP1 (HSP70 binding protein 1). The HSP110s are homologous to HSP70s and, despite lacking an ATPase-driven cycle, these co-chaperones are thought to bind substrate, differentiating them from the other NEF families (Höhfeld and Jentsch, 1997; Takayama and Reed, 2001; Chung et al., 2002; Kabani et al., 2002; Goeckeler et al., 2008; Xu et al., 2012; Kampinga and Craig, 2010).

A third class of co-chaperones contacts HSC70 or HSP70 through tetratricopeptide-repeat (TPR) adaptor domains; however, these do not directly affect the chaperone ATPase cycle (Young et al., 2003). The most relevant for our discussion is the C-terminus of HSP70-interacting protein CHIP (*STUB1*), which has E3 ubiquitin ligase activity and promotes proteasomal degradation of HSC70- or HSP70-bound substrates (Connell et al., 2001; Meacham et al., 2001). CHIP is soluble, whereas other ERAD-related E3 ligases are membrane-integrated and do not interact directly with the HSP70 chaperones (Fig. 1B). Polyubiquitylation is the first step in ERAD, followed by extraction of membrane proteins, unfolding and transfer to proteasomes, where degradation occurs (Smith et al., 2011; Hampton and Sommer, 2012). Another TPR co-chaperone is the HSP-organizing protein HOP (*STIP1*), which links HSC70 and HSP70 to the HSP90 chaperones so that the latter can complete chaperone-assisted folding (Fig. 1B). The human HSP90 chaperones, HSP90α (*HSP90AA1*) and HSP90β (*HSP90AB1*) are unrelated to HSP70s but are highly similar to each other, with shared ATP-dependent mechanisms and co-chaperones (Pearl and Prodromou, 2006). When referring to studies that could not distinguish between the different chaperone forms or which suggested equal involvement, the chaperones will be referred to as HSC/HSP70 or HSP90.

## CFTR: a paradigm for channel-trafficking defects

### Folding of CFTR

**Fig. 2.**
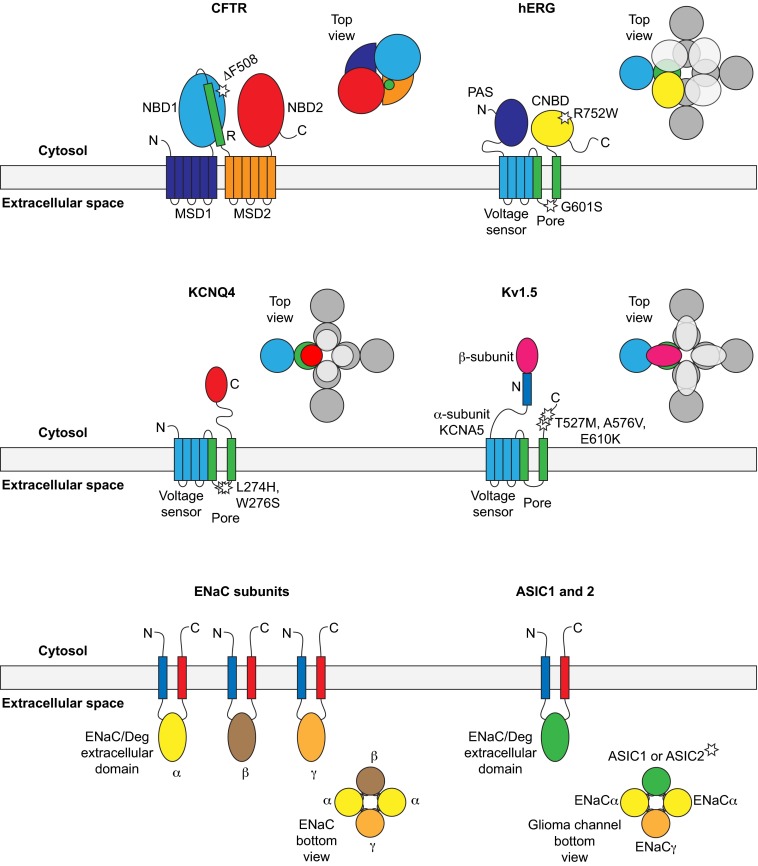
**Topologies of channels, oligomeric arrangement and trafficking-disease mutations**. Known or predicted transmembrane, cytosolic and extracellular domains are shown schematically, and the position of disease mutations discussed in this Review are marked with stars. CFTR contains two membrane-spanning domains (MSD1 and MSD2), two cytosolic nucleotide-binding domains (NBD1 and NBD2) and a regulatory region (R, to give NBD1-R). hERG, KCNQ4 and the Kv1.5 α-subunit KCNA5 have a conserved transmembrane region containing voltage-sensor and pore segments, and all form tetramers. In the top views, one subunit is shown in a color matching the domains, the other subunits in gray. hERG has a Per-Arnt-Sim (PAS) domain in its N-terminus and a cyclic-nucleotide-binding domain (CNBD) in its C-terminus, which likely also tetramerizes; the arrangement of the PAS domain is unclear and not shown in the top view. The KCNQ4 C-terminus contains a tetramerization domain. The KCNA5 N-terminus is the binding site for various β-subunits, which also form tetramers. The ENaC and ASIC subunits have a conserved structure including the ENaC/Deg (Degenerin) extracellular domain. The bottom view of the ENaC channel shows the expected α_2_βγ arrangement. The bottom view of the glioma-specific channel shows a hypothetical arrangement based on comparisons with the ENaC channel. The vascular-smooth-muscle-cell ASIC channels are heterogenous and not represented here. The ASIC2 subunit causes trafficking defects in the glioma-specific and vascular-smooth-muscle-cell ASIC channels.

The CFTR ATP-dependent chloride channel is a 1480-residue monomer containing two membrane-spanning domains (MSD1 and MSD2, each with six transmembrane helices) alternating with two cytosolic nucleotide-binding domains, one of which is bound to a regulatory region (NBD1-R and NBD2; Fig. 2) (Mornon et al., 2009). It is the only protein in the ATP-binding cassette (ABC) transporter family that functions as an ion channel. The most prevalent CF-associated mutation, ΔF508, maps to NBD1 (Table 1). As mentioned above, the deletion causes misfolding, retention at the ER instead of trafficking to the PM, and ERAD (Ward et al., 1995; Du et al., 2005; Riordan, 2008; Lukacs and Verkman, 2012). Early inhibitor studies found that HSP90 is essential for trafficking of WT and ΔF508 CFTR (Loo et al., 1998). Soon after, HSC70 and one of its co-chaperones, DNAJA1, were found to associate with CFTR, and there was more of each bound to ΔF508 than to WT (Meacham et al., 1999), consistent with the misfolded mutant exposing more HSC70-binding sites. Experiments with purified proteins found that HSC70 and DNAJA1 could prevent aggregation of the isolated NBD1 (Table 2) (Meacham et al., 1999). These results suggested that chaperones are engaged in trying to refold mutant CFTR. Direct evidence of functional folding was recently provided by siRNA knockdown of DNAJA1, which led to increased misfolding and ERAD, reducing the levels of both WT and ΔF508 CFTR (Fig. 2B) (Grove et al., 2011). Furthermore, overexpression of HSP70 and the co-chaperone DNAJB1 induced modest improvements in trafficking and stabilization of ΔF508 CFTR (Choo-Kang and Zeitlin, 2001; Farinha et al., 2002). Further confirmation was provided by a recent study that showed, using quantitative mass spectrometry, that ΔF508 CFTR accumulates in stalled folding intermediates enriched, compared to WT, in bound HSC/HSP70, HSP90 and DNAJB1, in line with the defect in folding and trafficking (Coppinger et al., 2012).

**Table 1.**
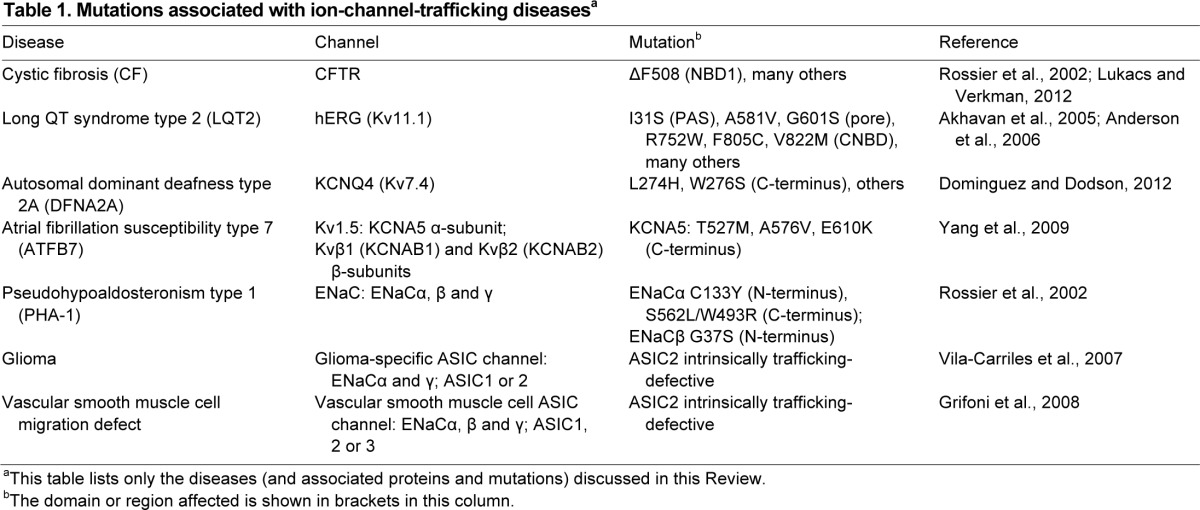
Mutations associated with ion-channel-trafficking diseases^a^

**Table 2.**
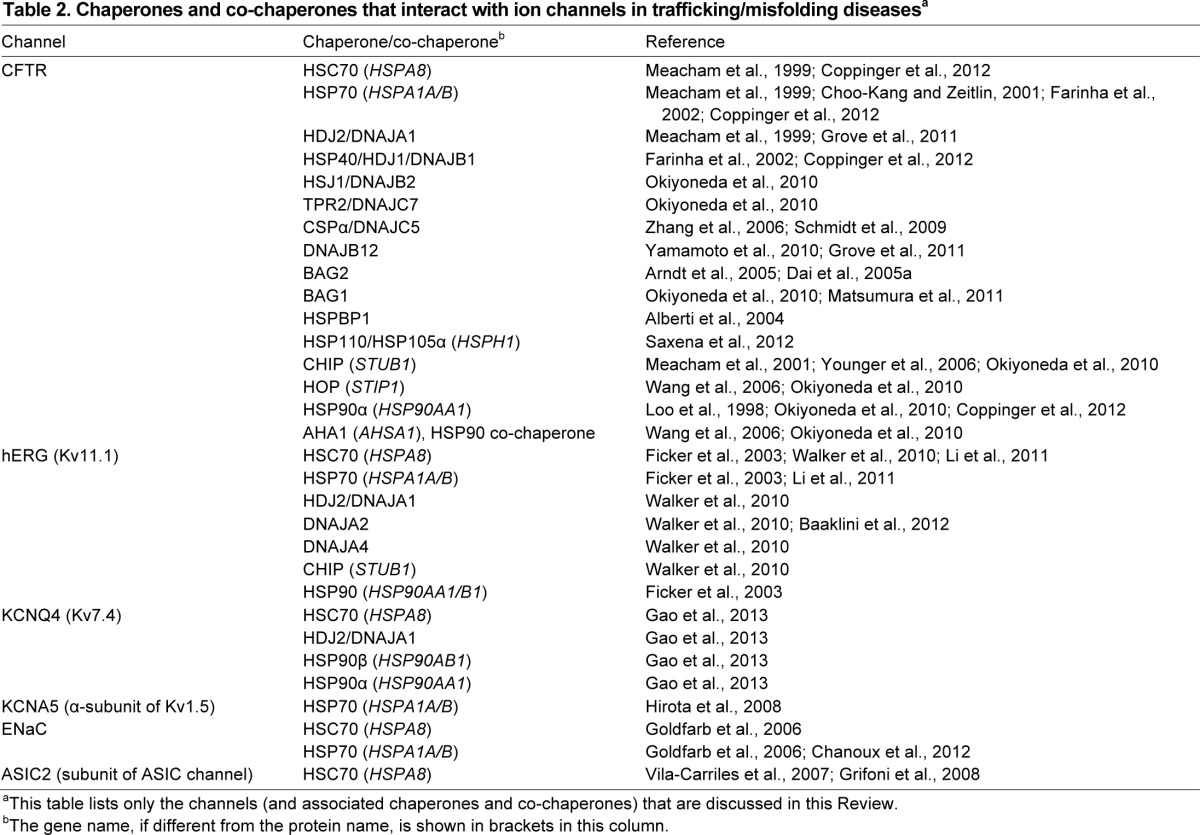
Chaperones and co-chaperones that interact with ion channels in trafficking/misfolding diseases^a^

### Co-chaperones and degradation

WT and ΔF508 CFTR were among the first proteins found to be degraded by the E3 ubiquitin ligase CHIP complexed with HSC/HSP70 (Meacham et al., 2001). As detailed above, CHIP is the only direct link known between the cytosolic chaperones and the ubiquitylation system. It acts in parallel to the ER transmembrane E3 ligases gp78 (*AMFR*) and RMA1 (*RNF5*) to select different regions of misfolded ΔF508 CFTR for degradation in the ER. HSC/HSP70-CHIP might recognize misfolding in the cytosolic domains, whereas gp78-RMA1 might be more sensitive to misfolding in the membrane (Fig. 2B) (Younger et al., 2006; Morito et al., 2008). Intriguingly, a parallel role for CHIP has recently been found at the PM. Expression of ΔF508 CFTR at low temperatures was observed to increase folding efficiency and allow some trafficking to the PM (Riordan, 2008). However, mutant CFTR at the PM is more rapidly internalized by endocytosis relative to WT CFTR, leading to degradation in the lysosomes. Knockdown siRNA screening and functional analysis identified CHIP as the E3 ligase responsible for initiating internalization by polyubiquitylating ΔF508 CFTR (Okiyoneda et al., 2010). Also, specific roles in internalization were identified for HSC70, DNAJA1, two other DNAJs – HSJ1 (DNAJB2) and TPR2 (DNAJC7) – the NEF BAG1, HSP90α, HOP and the HSP90-regulatory co-chaperone AHA1 (Table 2). Just as informative was the lack of involvement of closely related components tested in the screens: HSP70, DNAJB1, other NEFs and HSP90β; despite having related mechanisms within each family, the chaperones and co-chaperones have specific roles and are not interchangeable (Okiyoneda et al., 2010). The cell-surface role of CHIP was notable because it had been thought to act on secretory-pathway proteins only at the ER. It is interesting to note that some of the same components, particularly AHA1 (activator of HSP90 ATPase, *AHSA1*), were also found to act on ΔF508 CFTR at the ER (Wang et al., 2006). The ‘quality control’ chaperone systems at the ER and PM could thus work in parallel, although obviously in different cellular contexts. HSJ1 and TPR2 were not detected in the ER complexes and might have specific roles at the PM. HSJ1 binds polyubiquitylated proteins to promote their degradation (Westhoff et al., 2005), whereas TPR2 promotes transfer of substrates from HSP90 to HSC/HSP70 through its J domain and TPR domains (Brychzy et al., 2003), both in agreement with a central function of CHIP-HSC/HSP70 complexes in the degradation of mutant CFTR. This implies that this internalization pathway will need to be targeted as part of any therapy to maintain functional ΔF508 CFTR at the cell surface.

In addition to promoting folding of WT or ΔF508 CFTR by HSC/HSP70, a major function of DNAJs (Table 2) might in fact be to regulate degradation of ΔF508 CFTR. Cysteine string protein CSPα (also known as DNAJC5) is attached by lipid anchors on cysteine side chains to synaptic vesicles and other secretory pathway membranes, and has a role in regulating exocytosis and trimeric G-protein signaling (Johnson et al., 2010). With regards to CFTR, CSPα was also found at the ER; its knockdown promoted trafficking of WT CFTR, and its overexpression impaired trafficking. CSPα that had a mutation in its HSC/HSP70-interacting J domain was reported to have no effect on trafficked, mature CFTR but caused some accumulation of the immature form (Zhang et al., 2006). A subsequent study showed that CSPα overexpression also enhances ERAD of CFTR by CHIP and HSC/HSP70 (Schmidt et al., 2009).

Recently, DNAJB12 was discovered by two groups as an ER-transmembrane protein that interacts with HSC70. DNAJB12 overexpression promoted ERAD of WT and ΔF508 CFTR as well as certain other proteins, whereas knockdown of DNAJB12 protected ΔF508 CFTR from degradation, without affecting trafficking (Yamamoto et al., 2010; Grove et al., 2011). One group further showed that DNAJB12 enhances ΔF508 CFTR interaction with RMA1 and gp78, but, interestingly, not with CHIP, which would have been expected because of DNAJB12 stimulation of HSC70 binding. However, ΔF508 CFTR trafficking was improved by DNAJB12 knockdown combined with another stabilizing V510D mutation within CFTR and a small-molecule stabilizer, Corrector-4 (Grove et al., 2011). Co-chaperone-induced ERAD might be a substantial obstacle to the rescue of ΔF508 CFTR trafficking, but these last results imply that both pro-folding and anti-degradation strategies will be needed therapeutically. It is possible that CSPα and DNAJB12 interact with CFTR primarily through their membrane localization (Fig. 1B), but it remains to be determined how CSPα but not DNAJB12 functions through CHIP.

### NEFs regulate degradation

NEFs associated with CFTR (Table 2) are also involved in the intricate regulation of its degradation. BAG1 and BAG2 (*BAG2*) are similar in terms of biochemical effects on HSC/HSP70; however, only for BAG2 has it been shown that overexpression increases WT and ΔF508 CFTR levels by inhibiting CHIP, and its knockdown permits degradation of CFTR (Fig. 1B) (Arndt et al., 2005; Dai et al., 2005a). A recent report used reconstituted *in vitro* experiments to show that BAG1 does also impair HSC/HSP70-assisted CFTR folding and CHIP-mediated degradation. Although the excess of BAG1 required for optimal effects was difficult to achieve in cells, it was shown that its effects are mediated through its NEF mechanism, which promotes the dissociation of HSC/HSP70 from substrates (Fig. 1A,B), rather than through direct interactions with CHIP (Matsumura et al., 2011).

Another NEF, HSPBP1, binds CHIP directly and protects WT and ΔF508 CFTR from degradation by CHIP when overexpressed, and impairs CFTR trafficking on knockdown (Fig. 1B) (Alberti et al., 2004). HSP110 (also known as HSP105α; encoded by *HSPH1*) is one of the most recent NEFs identified; its knockdown reduced co-translational degradation of both WT and ΔF508 CFTR, and its overexpression promoted degradation while also leading to a proportional increase in ΔF508 CFTR trafficking. Moreover, the study showed that HSP110 associates with ΔF508 CFTR after trafficking and prolongs the lifetime of the mature state (Saxena et al., 2012). HSP110 is homologous to HSP70 chaperones and, although it does not have an ATP-dependent folding function (Kampinga and Craig, 2010; Young, 2010), its potential substrate-binding activity might be responsible for stabilization of mature ΔF508 CFTR. Thus, both the DNAJ and NEF co-chaperones influence the degradation aspect of HSC/HSP70, but through different mechanisms that warrant further research. There is growing evidence that closely related co-chaperones have different effects (DNAJB1 and DNAJB2, BAG1 and BAG2), and pharmacological targeting of individual proteins instead of entire families might be a more productive approach. Also, targeting the co-chaperones provides more options for enhancing the chaperone-mediated folding of mutant CFTR than targeting HSC70 or HSP70 itself. As suggested above, a combined strategy of inhibiting degradation and promoting folding will likely be needed. Thus, a more thorough understanding of chaperone mechanisms with CFTR is called for.

## hERG: an emerging paradigm of misfolding and trafficking

### Folding of hERG

The hERG (Kv11.1) voltage-gated potassium channel functions as a tetramer, minimally consisting of four identical subunits. Each 1159-residue subunit contains a cytosolic N-terminal region with the Per-Arnt-Sim (PAS) domain that is found in a variety of proteins: a membrane-spanning region with six transmembrane helices, and a cytosolic C-terminal region with a cyclic-nucleotide-binding domain (CNBD). The transmembrane region contains voltage-sensing and pore-forming segments, and is conserved in the voltage-gated potassium channel (Kv) superfamily; however, the cytosolic domains are not conserved between Kv channels (Fig. 2) (Sanguinetti and Tristani-Firouzi, 2006; Brelidze et al., 2013). A large number of LQT2 mutations in hERG are known, found throughout all regions of the polypeptide. Dominant inheritance arises from haploinsufficiency due to loss of function of the mutant hERG, or from dominant-negative effects of the mutant on WT hERG polypeptides when mixed tetramers are formed (Sanguinetti and Tristani-Firouzi, 2006). Many LQT2 mutations act by impairing hERG folding and trafficking, resulting in accumulation of the mutant protein at the ER (Table 1). One mutation in the pore domain, A561V, was first found to inhibit WT hERG trafficking in a dominant-negative manner, and other mutations are thought to act via the same mechanism (Ficker et al., 2000; Akhavan et al., 2005; Anderson et al., 2006).

The activity of pharmacological chaperones – drugs that specifically stabilize protein structure – on hERG mutants suggests that the equilibrium between misfolded and native states can be shifted towards the folded forms. These drugs (e.g. astemizole, E-4031) specifically bind to the transmembrane pore of WT hERG and block channel function. However, the drugs also bind many of the trafficking-defective LQT2 hERG forms mutated in the pore-forming region, to stabilize them and partially rescue their trafficking. In contrast, the drugs have no effects on trafficking-defective hERG with mutations in its independently folded N- or C-terminal regions (Anderson et al., 2006; Gong et al., 2006; Harley et al., 2012).

Manipulation of cellular chaperones could also shift the equilibrium towards increased folding, without the drawback of channel inhibition. An early study identified HSC/HSP70 and HSP90 in complexes with immature WT hERG at the ER (Table 2). HSP90 inhibition impaired hERG trafficking, and HSP70 overexpression increased overall hERG levels moderately. Both HSC/HSP70 and HSP90 remained associated with the trafficking-deficient LQT2 mutants R752W (mapping to the CNBD) and G601S (mapping to the pore), and decreased chaperone association with each mutant was observed upon rescue of trafficking by reducing the temperature or administering the pore-blocker astemizole, respectively (Ficker et al., 2003). The challenge for therapeutic development will be to take advantage of the folding activity of the chaperone system while diminishing its degradation role, as described above for cystic fibrosis.

### Degradation versus folding: striking the right balance

DNAJA1 is closely related to DNAJA2 and DNAJA4, although they have functional differences (Bhangoo et al., 2007). A recent study examined the role of these co-chaperones with regards to hERG trafficking (Table 2). Overexpression of each of these three co-chaperones individually was shown to inhibit WT hERG trafficking, owing to increased ERAD by CHIP; of these, DNAJA2 had the strongest effect (Walker et al., 2010). However, knockdown of DNAJA1 alone impaired hERG trafficking. This indicates that DNAJA1 is required to assist hERG folding by HSC/HSP70, and the endogenous levels of DNAJA1 seem to be optimized for folding; DNAJA2 and DNAJA4 might be less important for hERG folding. DNAJA2 impaired trafficking of G601S hERG but not WT hERG in low-temperature rescue conditions, suggesting that its degradation role is related to the physical stability of hERG. On the basis of this finding, it was therefore proposed that DNAJA2 and DNAJA4 act mainly as regulators of hERG degradation (Walker et al., 2010). A subsequent mechanistic study on DNAJA2 found that the protein is required instead of DNAJA1 for the HSC/HSP70-mediated folding of another model substrate, heat-denatured luciferase. Folding-inactive DNAJA2 mutants were also unable to promote hERG degradation, suggesting that DNAJA2-dependent folding and degradation are in fact closely related mechanistically (Baaklini et al., 2012). Similar to the issue with HSC/HSP70, a next question will be how to separate the pro-folding and degradation roles of these DNAJs.

A comparison of HSP70 and HSC70 functions in hERG folding and stability found opposite effects (Table 2). Knockdown of HSP70 reduced overall levels of WT hERG, but knockdown of HSC70 increased hERG levels. Opposite to HSP70, overexpression of HSC70 decreased hERG levels (Li et al., 2011). The differences with respect to HSC70 or HSP70 overexpression were found to be due to differences in levels of degradation of immature hERG at the ER, which affected the amounts of functional hERG channel at the PM. On comparing a panel of LQT2 mutants that had WT hERG, mutations in the cytosolic domains resulted in increased binding by HSC70, but decreased binding by HSP70; conversely, some mutations in the pore region decreased HSC70 binding (Li et al., 2011). The latter results provide another clue that the biological effects of the chaperone system depend on the specifics of the hERG folded state and chaperone/co-chaperone mechanisms. Furthermore, it is possible that combined manipulation of HSC70, HSP70 and DNAJA1 could enhance their assistance of hERG folding, for at least some of the LQT2 mutants.

## Other channels: potential new paradigms

### Voltage-gated potassium channels

The KCNQ4 (Kv7.4; encoded by *KCNQ4*) voltage-gated potassium channel is in the Kv protein superfamily. It assists in the transmission of signals in the auditory neural sensory pathway, by regulating ion flux in the cochlea. The 695-residue polypeptide forms homotetramers and has the conserved Kv transmembrane region but a cytosolic C-terminal region that also tetramerizes (Fig. 2) (Howard et al., 2007; Dominguez and Dodson, 2012). Twelve known missense mutations in the KCNQ4 transmembrane region lead to autosomal dominant deafness type 2A (DFNA2A; Table 1) with late-onset progressive hearing loss. The genetic mechanism is thought to be haploinsufficiency, although some mutants act to dominantly inhibit WT KCNQ4 in heterotetramers. Certain mutants show trafficking defects, which might be the cause of channel loss-of-function (Kharkovets et al., 2006; Kim et al., 2011; Dominguez and Dodson, 2012). In a recent analysis of chaperones, knockdown and overexpression showed that HSP90β, HSC70 and DNAJA1 enhance KCNQ4 levels, consistent with increased folding; however, HSP90α diminished KCNQ4 levels (Table 2). HSP90β overexpression restored PM expression of the DFNA2A-trafficking-defective pore mutants L274H and W276S, whereas DNAJA1 and HSP70 had a small effect. In addition, HSP90β increased the number of active WT KCNQ4 channels at the cell surface, and slightly that of WT and L274H mixed channels; the number of active WT and W276S mixed channels was not improved (Gao et al., 2013). The reason for this difference is unknown, but it might suggest that chaperones are sensitive to relatively small changes in protein conformation. The link between folding and PM trafficking of KCNQ4 and its mutants will require further study to shed light on other co-chaperones and the mechanisms of ERAD.

The Kv1.5 delayed rectifier potassium channel α-subunit (*KCNA5*) is also in the Kv superfamily; like hERG, it acts in repolarization of cardiac muscle, but its expression is restricted to the atrium. The 613-residue KCNA5 polypeptide has cytosolic N-and C-terminal regions flanking its transmembrane region; four cytosolic β-subunits (isoforms of Kvβ1 and Kvβ2, encoded by *KCNAB1* and *KCNAB2*, respectively) assemble onto the N-termini of KCNA5 homotetramers, to form the complete Kv1.5 channel (Fig. 2) (Ravens and Wettwer, 2011). A nonsense mutation in the pore domain (E375X) and three missense mutations in the C-terminal region of KCNA5 (T527M, A576V, E610K) are associated with autosomal dominant atrial fibrillation susceptibility type 7 (ATFB7; Table 1). The mutants act as dominant-negative inhibitors of WT KCNA5, and the partial activity of the missense mutants is consistent with probable folding defects (Olson et al., 2006; Yang et al., 2009). WT Kv1.5 has previously been shown to be unaffected by HSP90 inhibition (Ficker et al., 2003). A more recent study found that overexpression of HSP70 enhanced Kv1.5 levels, consistent with increased folding and trafficking, whereas DNAJB1 had no effect (Hirota et al., 2008); as with CFTR and hERG, other DNAJs might be more active. Overexpressed HSP70 furthermore prolonged the lifetime of the channel and increased its functional density at the cell surface (Hirota et al., 2008). Experiments with the ATFB7 mutants, as homo- or heterotetramers with WT KCNA5, and investigating β-subunit assembly are the logical next steps. Because the cytosolic regions of hERG, KCNQ4 and KCNA5 are so different from each other, it seems unlikely that their chaperone/co-chaperone requirements for folding are exactly identical. On the other hand, the mechanisms that recognize misfolding in their conserved transmembrane region might be similar; these questions remain to be addressed.

### ENaC/Degenerin cation channels

In the kidney, colon and lung, the epithelial sodium channel ENaC has a key role in maintaining serum sodium balance and blood pressure. ENaC contains three homologous subunits in an α_2_βγ stoichiometry (*SCNN1A*, *SCNN1B* and *SCNN1G*, respectively). The subunit polypeptides are between 640 and 669 residues long, and share a structure conserved in the ENaC/Degenerin family: cytosolic N- and C-terminal domains flanking two transmembrane helices and an extracellular domain (Fig. 2). Loss-of-function mutations in any of the three subunits, including missense mutations in ENaCα and ENaCβ that cause trafficking defects (Table 1), lead to salt-wasting pseudohypoaldosteronism type 1 (PHA-1), which is associated with hypotension and risk of pulmonary edema (Kellenberger and Schild, 2002; Rossier et al., 2002). An early report using a *Xenopus* oocyte expression system suggested that overexpression of human HSC70 decreased the expression of mouse ENaC as measured by cell-surface current, but HSP70 increased ENaC levels (Table 2) (Goldfarb et al., 2006). Although intriguing, interpretation of these findings was complicated by fundamental differences between oocytes and human cells, and the lack of information on oocyte chaperone systems. A recent study has now addressed the question in mammalian MDCK cells. Overexpression of moderate amounts of HSP70 was found to increase ENaC total expression and also levels at the PM, but higher amounts did not. The enhanced trafficking was attributed to increased assembly of the α- and β-subunits and interaction with the SEC24D component of the COPII anterograde machinery responsible for vesicle traffic out of the ER, in agreement with a folding role of HSP70. No changes were found in ENaC turnover at the PM. The lack of effect of high HSP70 levels was speculated to be due to disruption of mRNA turnover or transcription by excess HSP70 (Chanoux et al., 2012). It is possible that the effects of HSP70 on trafficking-deficient ENaC mutants are more pronounced.

Sodium and calcium channels containing the acid-sensing ion channel subunits ASIC1, ASIC2 and ASIC3 (*ASIC1*, *ASIC2* and *ASIC3*) are thought to function in neurotransmission. The subunits, which belong to the ENaC/Degenerin family, have a similar architecture to each other and are between 512 and 574 residues long. They have been proposed to form mixed tetramers with individual ENaC subunits to form the constitutively active amiloride-sensitive sodium channel found in glioma (but not untransformed astrocytes) (Fig. 2). More precisely, channels containing ASIC1 or ASIC2 together with ENaCα and ENaCγ are thought to form the active ASIC channels at the cell surface. Cells that have the lowest PM expression of the ASIC2 subunit, despite high intracellular levels, also have the highest channel activity and malignancy, suggesting a folding and trafficking defect (Vila-Carriles et al., 2007; Kapoor et al., 2011). HSC70, but not HSP70, was found to associate with ASIC2 in glioma cells, and HSC70 knockdown increased the cell-surface expression of ASIC2 and reduced the channel activity (Table 2). This treatment to increase levels of PM ASIC2 also inhibited glioma cell migration (Vila-Carriles et al., 2007).

The ASIC proteins also have important roles in regulating vascular smooth muscle cell migration: higher amiloride-sensitive channel activity in mixed heteromers with ASIC1, ASIC3 and separate ENaCα, ENaCβ and ENaCγ subunits corresponds to more migration. Only ASIC2 was mostly retained at the ER and associated with HSC70; glycerol, a non-specific chemical chaperone, increased total and PM ASIC2 levels, and inhibited cell migration, mimicking the glioma paradigm. Furthermore, HSC70 knockdown promoted PM levels of ASIC2, in primarily a truncated form, and strongly inhibited cell migration (Grifoni et al., 2008). In both these cases, the molecular lesion that causes the ASIC2 trafficking defect is unknown – in glioma it might be oncogenic mutations or modifications, whereas, in vascular smooth muscle cells, the truncated form could be involved. Because HSC70 functions to inhibit ASIC2 trafficking, it is interesting to speculate that its ERAD role with CHIP might be responsible. Furthermore, the ASIC sodium channel role in glioma could represent an adaptation in the cancer cells to favor proliferation and invasion, and the involvement of HSC70 might be part of a broader tumor-cell dependence on chaperones. HSP70, and in some cases HSC70, is important for tumor cell expansion through regulation of pathways including apoptosis and autophagy (Goloudina et al., 2012). HSC70 regulation of malignancy via the composition of the ASIC channel would be an additional mechanism.

## Outlook

The emerging picture of HSC/HSP70 in ion-channel-misfolding diseases is complex, but highlights the opposing functions at the ER of assisting folding and promoting degradation. The parallel functions at the PM are just beginning to be explored, but the specific mechanisms involved are likely to differ depending on the channel – in the case of ΔF508 CFTR, loss of CHIP activity at the ER is not sufficient to allow trafficking, whereas, at the PM, it is sufficient to impair internalization. Another point is the biological difference between HSC70 and HSP70, for which there is as yet no known biochemical basis. Some of the reported differences in knockdown results might be due to varying expression levels of HSC70 and HSP70 in different cell types, yet the differences observed when they are overexpressed (thus masking any differences in endogenous expression) suggest that there is something more at play. The percentage identity and similarity between HSC70 and HSP70 is remarkably high: 87% sequence identity and 94% similarity. However, it is possible that minor differences in structure, biochemical properties or post-translational modification result in significant functional divergence in cells. There might also be differences in the range of co-chaperones modulating HSC70 and HSP70; there is no evidence yet of interactions that are exclusive to HSC70 or HSP70, but it is very plausible that some co-chaperones interact preferentially with one or the other, owing to differing affinities and relative cellular concentrations. Regarding knockdowns, it should be noted for the relevant studies (Vila-Carriles et al., 2007; Grifoni et al., 2008; Okiyoneda et al., 2010; Li et al., 2011; Gao et al., 2013) that depletion of HSC70 induces HSP70 expression but not the other way around, and silencing of both is sometimes required for full effects (Powers et al., 2008).

Distinguishing the folding and degradation roles of HSC/HSP70 will be an important question to be addressed. The study of HSP70 and ENaC channel trafficking (Chanoux et al., 2012) did this by using HSP70 fused to an epitope tag at its C-terminus, disrupting the interaction site for CHIP as well as other TPR co-chaperones (Scheufler et al., 2000; Graf et al., 2010; Wang et al., 2011). A similar approach could be useful in future studies. The sometimes overlapping or opposing roles of the DNAJ and NEF co-chaperones, many still uncharacterized with respect to ion-channel-misfolding diseases, will need to be systematically addressed, with knockdowns as the primary tool, followed by overexpression. Either the folding or degradation roles of the chaperone system could be helpful in specific cases of dominant-negative inhibition of heteromeric channels. For example, the trafficking of hERG WT and A561V heterotetramers could be improved by enhanced chaperone-mediated folding of the mutant subunit cytosolic domains. Because the mutation itself lies in the transmembrane pore, the folding activity of chaperones might be limited. Instead, faster clearance of mutant subunits by enhanced chaperone-mediated degradation could permit more WT homotetramers to form. The problem of haploinsuffiency would still remain to be addressed therapeutically by measures to increase WT hERG expression, but this could be more feasible after reducing dominant-negative inhibition.

Recent reports of HSC/HSP70 inhibitors indicate a growing interest in this drug target, and effects on CFTR trafficking have now been reported. Purified HSP70 activity was inhibited by an adamantyl-modified form of the cellular lipid sulfogalactosyl ceramide (adaSGC). In cells, adaSGC increases trafficking of ΔF508 CFTR under temperature rescue conditions (Park et al., 2009). Although the use of adaSGC was complicated by changes in endogenous lipids, the study suggested that HSC/HSP70 was inhibitory of mutant-CFTR trafficking, most probably owing to CHIP-mediated degradation. Recently, an imidazole-based compound, apoptozole (Az), was identified in a screen for drugs that promoted apoptosis. It was found to interact with HSC/HSP70 in cells, and inhibited HSC70 *in vitro*. Az treatment rescued some trafficking and channel function of ΔF508 CFTR at 37°C, but had a lesser effect on internalization of the mutant protein from the cell surface. Moreover, Az interfered with ubiquitylation of ΔF508 CFTR by HSC/HSP70-CHIP complexes (Cho et al., 2011). Thus, the degradation versus folding function of HSC/HSP70 remains a key question. Other putative inhibitors have not yet been tested for effects on CFTR or hERG. As with Az, questions over the specificity of these drugs, as well as their mechanism of action (Leu et al., 2009) or efficacy in live cells (Chang et al., 2011), are expected to be resolved.

Better inhibitors of HSC/HSP70 will raise ion-channel-misfolding research to an entirely new level, and combined drug and knockdown screening of co-chaperones could develop into a powerful approach. Furthermore, pharmacological targeting of HSC/HSP70 or eventually its co-chaperones could open up prospects for novel therapies towards CF and LQT2, and the other diseases of protein misfolding and trafficking. A concern to be addressed will be pleiotropic effects of such therapies – for example, inhibition of HSP70 to relieve CHIP-mediated degradation of ΔF508 CFTR could also impair HSP70-dependent folding of hERG, leading to cardiac arrhythmias in CF patients. Some of these issues might be dealt with during drug testing for side effects, with prioritization of specific physiological systems that are likely to be affected. In some cases, enhancement of HSC/HSP70 activity might have greater positive effects on mutated channels than on WT channels in the affected individual. This can be compared with the development of HSP70 inhibitors as anti-cancer agents, with one rationale being that mutated oncoproteins are abnormally dependent on HSP70 – chaperone ‘addiction’ – and are more sensitive to HSP70 inhibition (Powers et al., 2008; Goloudina et al., 2012). The rationale is somewhat reversed in the genetic diseases: mutant channels would be more sensitive to improved chaperone activity. Finally, an ideal strategy would target the small molecule to the tissue where the genetic disease is most harmful. The specificity required is not currently feasible, but new advances in drug delivery approaches rather than the drugs themselves are likely to address this challenge. Thus, there are prospects for both inhibitors and stimulators of HSC/HSP70 function.
